# The Influence of Sex, Gender, or Age on Outcomes of Digital Technologies for Treatment and Monitoring of Chronic Obstructive Pulmonary Disease: Protocol for an Overview of Systematic Reviews

**DOI:** 10.2196/40538

**Published:** 2022-10-12

**Authors:** Katja Matthias, Ivonne Honekamp, Karina Karolina De Santis

**Affiliations:** 1 Faculty of Electrical Engineering and Computer Science University of Applied Science Stralsund Stralsund Germany; 2 Faculty of Business and Economics University of Applied Science Stralsund Stralsund Germany; 3 Department of Prevention and Evaluation Leibniz Institute for Prevention Research and Epidemiology - BIPS GmbH Bremen Germany

**Keywords:** digital technologies, digital intervention, COPD, AMSTAR 2, chronic obstructive pulmonary disease, gender, sex, age, overview, systematic review, treatment, monitoring, chronic disease, chronic illness, outcome reporting, review methodology, critical appraisal

## Abstract

**Background:**

Chronic obstructive pulmonary disease (COPD) is a common chronic disease that can be treated and monitored with various digital technologies. Digital technologies offer unique opportunities for treating and monitoring people with chronic diseases, but little is known about whether the outcomes of such technologies depend on sex, gender, or age in people with COPD.

**Objective:**

The general objective of this study is to assess the possible influence of sex, gender, or age on outcomes of digital technologies for treatment and monitoring of COPD through an overview of systematic reviews.

**Methods:**

The study is planned as an overview of systematic reviews. Study reporting is based on the PRISMA (Preferred Reporting Items for Systematic reviews and Meta-Analyses) 2020 guidelines because guidelines for overviews are not available as of this writing. The information sources for the overview will include 4 bibliographic databases (MEDLINE, Cochrane Library, Epistemonikos, and Web of Science) as well as the bibliographies of the included systematic reviews. The electronic search strategy will be developed and conducted in collaboration with an experienced database specialist. The search results will be presented in accordance with the PRISMA 2020 guidelines. The eligibility of studies is based on the population, intervention, comparison, outcomes, and study design (PICOS) criteria: (1) people with COPD (population), (2) digital technology intervention for treatment or monitoring (intervention), (3) any control group or no control group (comparison), (4) any outcome, and (5) systematic review of randomized controlled trials or non–randomized controlled trials with or without a meta-analysis (study design). Critical appraisal of the included systematic reviews will be performed using A Measurement Tool to Assess Systematic Reviews, version 2 (AMSTAR 2). Data will be extracted using a standardized data extraction sheet.

**Results:**

The literature search is scheduled for June 2022. We expect to select the relevant systematic reviews, code the data, and appraise the systematic reviews by December 2022.

**Conclusions:**

There is a growing recognition that the influence of sex, gender, or age should be considered in research design and outcome reporting in the context of health care interventions. Our overview will identify systematic reviews of various digital technologies for treatment or monitoring of COPD. The most interesting aspect of the overview will be to investigate if any systematic reviews considered the influence of sex, gender, or age on the outcomes of such digital technologies in COPD. Evidence from the overview could be used to guide more individualized (sex, gender, or age-based) recommendations for the use of digital technologies among people with COPD.

**Trial Registration:**

PROSPERO International Prospective Register of Systematic Reviews CRD42022322924; https://www.crd.york.ac.uk/prospero/display_record.php?RecordID=322924

## Introduction

Chronic obstructive pulmonary disease (COPD) is a prevalent chronic disease associated with a high disease burden and premature death [1,2]. The prevalence increases with age [3], and differences in diagnostic and therapeutic responses depending on sex, gender, or age have been found [4-12]. For example, although females manifest more severe COPD symptoms across their life course than males [9], they also benefit to a greater extent from certain therapeutic interventions [10]. Female sex is also associated with severe early-onset COPD [11].

In general, sex and gender appear to be inconsistently defined in the literature on COPD. Some studies refer exclusively to sex [10-12], others exclusively or predominantly to gender [4,5], and some use both terms [8,9]. We refer to “sex” as a genetic or biological construct that distinguishes between males and females and to “gender” as a social construct [2,13]. Despite these distinctions, sex and gender cannot be neatly separated because the concepts are multidimensional and interrelated [13]. It is increasingly understood that sex-specific biological factors and social factors influence each other and interact to affect health behaviors, opportunities, and outcomes [8]. Owing to such complexity of definitions, we aim to use any definition of sex or gender used in the context of COPD.

Digital technologies offer unique opportunities for treatment and monitoring of people with chronic diseases [14-17]. Digital technologies can help shift from reactive to proactive treatment approaches [18], but it is known that the uptake of digital technologies varies and depends, among other factors, on sex, gender, or age [19,20].

In recent years, many systematic reviews have been published on the use of digital technologies in COPD. If methodologically sound systematic reviews on a similar topic already exist, a new method of research synthesis, a so-called overview (a systematic review of systematic reviews) [21], can be conducted. Overviews can summarize the outcomes of multiple systematic reviews with similar objectives and address new objectives using existing data reported in such reviews. Although compared to systematic reviews, the number of overviews is still relatively low, the popularity of the latter is growing exponentially [22]. The main difference between an overview and a systematic review is that the units of searching, inclusion, and data analysis are systematic reviews (in overviews) and primary studies (in systematic reviews).

Systematic reviews should provide a comprehensive and objective assessment of existing evidence. This includes appropriate consideration of sex, gender, or age differences in the outcomes of any health care intervention. It is unclear if and to what extent systematic reviews have thus far addressed the influence of sex, gender, or age on the outcomes of digital technologies for treatment and monitoring of COPD. According to a search of the International Prospective Registry of Systematic Reviews (PROSPERO), MEDLINE, and the Cochrane Database of Systematic Reviews, no currently planned or completed overviews of systematic reviews on this topic were identified.

Thus, our main objectives are to (1) describe the terminology and definitions of sex or gender used in the systematic reviews; (2) determine if the systematic reviews focus on sex, gender, or age in any planned analyses and result reporting; (3) assess whether the systematic reviews include sex, gender, or age in their implications for clinical practice or policy and regulation development; and (4) create an evidence map that could inform individualized recommendations for people with COPD that take into account sex, gender, or age.

## Methods

### Study Design

The study is planned as an overview of systematic reviews [21]. Study reporting is based on the PRISMA (Preferred Reporting Items for Systematic reviews and Meta-Analyses) 2020 guidelines [23] because guidelines for overviews are not available at the time of this writing. However, a new set of guidelines (Preferred Reporting Items for Overviews of Reviews [PRIOR]) is expected to be published shortly [24]. Since the wording and structure of the PRIOR items resemble those of the PRISMA 2020 items, we intend to adhere to the PRIOR statement once it is published. The PRISMA 2020 or the PRIOR checklist will be made available once the overview is complete.

### Protocol and Registration

The overview of systematic reviews was prospectively registered on PROSPERO (CRD42022322924). Any changes to the protocol will be amended in PROSPERO and reported once the overview is complete.

### Patient and Public Involvement

Patients and the public were not involved in the design of this protocol. Thus, ethics approval is not required for the overview of systematic reviews.

### Eligibility Criteria

The eligibility criteria for this overview of systematic reviews are based on the population, intervention, comparison, outcomes, and study design (PICOS) criteria (Textbox 1). Our overview aims to (1) identify systematic reviews of digital technologies for the treatment and monitoring of COPD and (2) systematically assess if the outcomes reported in such reviews were analyzed or discussed in terms of sex, gender, or age. Consequently, we shall include neither the terms “sex,” “gender,” or “age” among the inclusion or exclusion criteria nor the search terms because we are interested in both types of systematic reviews in this field (ie, systematic reviews that either consider or do not consider the influence of sex, gender, or age on their outcomes).

We intend to include only systematic reviews in the languages in which we are proficient (English and German). We will report the number of systematic reviews that were excluded in the full-text screening owing to language considerations and discuss any possible implications of excluding such literature on the results of the overview.

**Eligibility criteria for the overview of systematic reviews.**

**Inclusion criteria**

- Population: diagnosis of chronic obstructive pulmonary disease (COPD) with or without any comorbidities
- Intervention: any digital technology for treatment and monitoring of COPD. Digital technologies are defined as any intervention delivered or supported by digital tools with the aim of targeted client communication or personal health tracking [25]; for example, remote and Web 2.0–based interventions that provide patients access to eHealth information regarding behavior change for self-management of COPD
- Comparison: any other intervention or no intervention
- Outcome: any outcome
- Study type: systematic review of randomized controlled trials (RCTs), non-RCTs, or both with or without meta-analysis. A study will be classified as a systematic review if it has explicitly stated objectives and reproducible methodology, including a literature search in at least 2 bibliographic databases
- Publication status: systematic review published in a peer-reviewed journal
- Publication language: English or German
- Full text accessible

**Exclusion criteria**

- Population without COPD
- Digital interventions are not applied or are not the primary intervention
- Other study type: rapid, scoping, or narrative review; overview of systematic review; primary study; comment; correction; letter; editorial; or protocol
- Other publication status: conference paper, unpublished report, thesis, or book
- Language other than English or German
- A review that does not fulfill the requirements for a systematic review (eg, no explicitly stated objectives or reproducible methodology or a literature search in only one bibliographic database) or has low or critically low appraisal ratings on AMSTAR 2 (A Measurement Tool to Assess Systematic Reviews, version 2) [26]
- Full text not accessible

### Information Sources

The information sources for the overview will include 4 bibliographic databases (MEDLINE, Cochrane Library, Epistemonikos, and Web of Science) as well as the bibliographies of the included systematic reviews. These databases were selected because they identified the most relevant studies in our preliminary search for systematic reviews and were accessible at our institution.

### Search Strategy

The electronic search strategy will be developed iteratively by the team in consultation with an experienced database specialist. The development and reporting of the search strategy adheres to the Peer Review of Electronic Search Strategies [27] and PRISMA Statement for Reporting Literature Searches in Systematic Reviews [28] guidelines. The search terms and corresponding Medical Subject Headings terms will be derived to address the 2 main search topics: (1) COPD and (2) digital technologies. The electronic search will be conducted in English by the first author and will not use any restrictions regarding language or time frame. We will use an incorporated and validated filter in MEDLINE to identify systematic reviews [29]. A summary of the electronic search in MEDLINE is shown in Table 1.

**Table 1:** Summary of the search strategy in MEDLINE.

Variable	Search topic 1: digital technologies	Search topic 2: chronic obstructive pulmonary disease
Example search terms	Telemed*, telehealth*, ehealth*, mhealth*, mobile applications, wearable electronic devices, digital*, healthcare application*, internet*	Chronic obstructive pulmonary disease*, chronic obstructive airways disease*, COPD, COAD
Search fields	Titles or abstracts	Titles or abstracts
Comments	Relevant Medical Subject Headings terms were included	Relevant Medical Subject Headings terms were included

### Selection of Sources of Evidence

**Figure 1:**
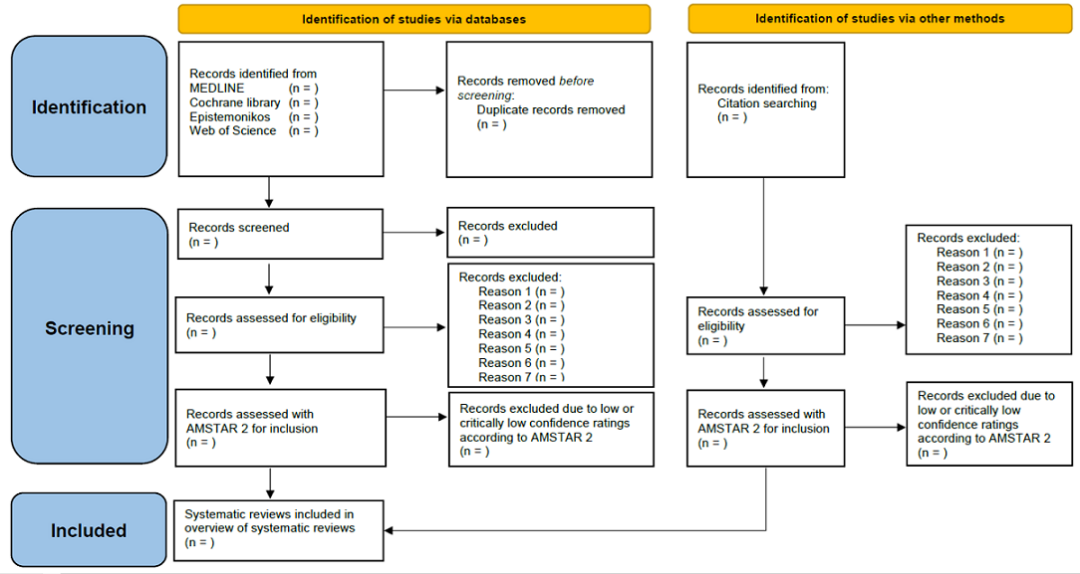
Modified Preferred Reporting Items for Systematic Reviews and Meta-Analyses (PRISMA) 2020 flow diagram.

The electronic search results will be stored in EndNote 20 (Clarivate). Following the removal of duplicates in EndNote, the remaining studies will be screened by 2 authors for inclusion in 3 steps using Covidence (Veritas Health Innovation). First, 2 authors will independently screen all titles and abstracts and reach consensus by discussion. Second, 2 authors will independently screen the studies selected for full-text inspection and reach consensus through discussion. In the case of no consensus, a third author will intervene. Third, once the study selection from the electronic search is complete, all systematic reviews will be appraised with AMSTAR 2 [26], and any systematic reviews with low or critically low appraisal ratings will be excluded owing to poor confidence in their results. One author will also manually screen the bibliographies of the included systematic reviews for additional literature. The results of the literature search will be reported in full once the overview is complete and presented on a PRISMA 2020 flow diagram [23] modified in accordance with our eligibility criteria and screening procedure (Figure 1).

A list of included and excluded studies following full-text screening and individual reasons for exclusion will be reported once the overview is complete.

### Critical Appraisal of Individual Sources of Evidence

The critical appraisal of systematic reviews will be performed using AMSTAR 2 [26]. AMSTAR 2 has acceptable psychometric properties and is an appropriate tool to appraise systematic review of health care interventions [26,30]. The tool includes 16 items that need to be rated to derive the overall confidence rating in the results of a systematic review (critically low, low, moderate, or high) [26]. The overall confidence rating will be derived for each systematic review on the basis of a combination of scores on 7 critical and 9 noncritical items in accordance with AMSTAR 2 guidelines [26].

A form for appraising systematic reviews with AMSTAR 2 will be self-developed in Excel (version 10; Microsoft Corp). AMSTAR 2 appraisals will be performed in 2 phases independently by 2 authors as described in our protocol for a scoping review [31] and consensus will be reached through discussion. In the case of no consensus, a third author will intervene. The overall confidence rating for each systematic review according to AMSTAR 2 will be reported once the overview is complete.

### Overlap in Primary Studies Included in Systematic Reviews

An overlap in overviews occurs when the same primary studies are cited in 2 or more systematic reviews. We will determine the overlap among primary studies in the included systematic reviews. Although there is currently no standardized methodological approach for addressing overlap in overviews [32], the creation of citation matrices and the calculation of the overall corrected covered area (CCA) can be used to visualize the overlap. In general, CCA refers to an overall degree in overlap in primary studies among all systematic reviews and can be computed using the Graphical Representation of Overlap for OVErviews tool [33]. The primary studies included in each systematic review will be inserted into this tool and compared among the systematic reviews (sorted from the oldest to the newest). The tool reports the absolute number of overlapped and nonoverlapped primary studies and an overall outcome of the CCA assessment (degree of overlap in the overview) [33].

### Data Charting

A form for coding and capturing of all data will be self-developed in Excel and calibrated within the team. Part of the data charting form will be adapted from the Sex and Gender Equity in Research guidelines [34]. Two authors will code all data independently in a 10% sample of the included systematic reviews. If the agreement in the sample is high (ie, reaching a κ of ≥0.80), the data in remaining systematic reviews will be charted by 1 author. We will resolve any discrepancies through discussion. In the case of no consensus, a third author will intervene. We will not contact the authors of the systematic reviews to obtain missing information or further clarification.

### Data Items

Data items that will be coded in the overview are reported in Textbox 2. These items were chosen to address the objectives of our overview. Data items will include descriptive characteristics of the systematic reviews and their included primary studies and any sex, gender, or age effects on any intervention outcomes. Data items (Textbox 2) will be coded either quantitatively into predefined categories or qualitatively using definitions or author statements from the included systematic reviews. All data will be reported once the overview is complete.

**Data items in the overview of systematic reviews.**

**Data items**

- Bibliographic information
- Population characteristics
- Intervention details
- Comparison type
- Outcome type
- Study (systematic review) type: Cochrane or non-Cochrane review
- Study aim according to review authors
- Primary studies in systematic review (number of studies, designs, and overlap among published studies)
- Risk of bias in primary studies according to review authors
- Data items for sex, gender, or age (eg, sensitivity analyses of outcomes taking into account sex, gender, or age)

### Synthesis of Results

The data will be synthesized using descriptive statistics (absolute frequencies) or narratively. The overall confidence ratings for all systematic reviews, obtained using AMSTAR 2, will be graphically synthesized using a bar graph to visualize the outcomes of the critical appraisal.

### Subgroup Analyses

Subgroup analyses will be performed to assess if considerations of sex, gender, or age in a systematic review are associated with the type or AMSTAR 2 appraisal rating of systematic reviews in accordance with methods applied in our previous work [22]. Proportions of studies with sex, gender, or age considerations (yes or no) will be compared on the basis of (1) the type of systematic review (Cochrane vs non-Cochrane) and (2) AMSTAR 2 confidence rating (high vs moderate) using chi-square tests and odds ratios with 95% CIs. These analyses will be performed because Cochrane reviews are associated with a higher quality than non-Cochrane reviews [35] and because high AMSTAR 2 ratings indicate high confidence in the results of a systematic review.

## Results

The literature search is scheduled for June 2022. We expect to select the relevant systematic reviews, code the data, and appraise the systematic reviews by December 2022.

## Discussion

### Principal Findings

Preliminary literature searches have shown that systematic reviews so far identified various digital technologies for the treatment or monitoring of COPD, including remote and Web 2.0–based interventions, internet-based telecommunication with health care professionals, telerehabilitation, smartphone interventions, and home telemonitoring. The overview will provide a detailed list of such technologies once the studies are selected. We will also assess the outcomes of such digital technologies in the context of COPD. The most interesting aspect of the overview will be to investigate if any systematic reviews have considered sex, gender, or age in their data synthesis or discussion of outcomes of such digital technologies in COPD.

### Comparison to Prior Work

There is a growing recognition of the importance of sex, gender, or age considerations in research design and reporting [36-39]. This applies to not only primary studies but also systematic reviews. This can be challenging because the use of multiple subgroup analyses can cause methodological problems [40,41]. Methodological studies assessing the consideration of sex or gender, mostly included in Cochrane reviews, show room for improvement [42-45]. A recent methodological study evaluating a sample of 113 Cochrane reviews of interventions to prevent health care–associated infections found that only 10 reviews (10%) planned to conduct a subgroup analysis based on sex and only 3 (3%) reported the results of such an analysis [45]. It remains unclear whether this is also an issue with systematic reviews of digital technologies for COPD. According to the literature identified in the context of preparing this protocol, we have noticed that the terms “sex” and “gender” are not used in a standardized way in studies on COPD [4,5,8-12]. This is consistent with the findings of Adisso et al [46], who conducted a secondary analysis of a Cochrane systematic review that assessed sex and gender terminology in shared decision-making studies. Adisso et al [46] concluded the following:

In SDM implementation studies, sex and gender terms and concepts are in a state of confusion. Our results suggest the urgency of adopting a standardized use of sex and gender terms and concepts before these considerations can be properly integrated into implementation research.

Thus, our overview will provide all terminology and definitions of sex and gender used in the systematic reviews of digital technologies for COPD.

Our overview focuses on the potential influence of only 3 sociodemographic variables (sex or gender and age) on the outcomes of digital technologies in COPD. We assume that these variables are regularly collected and reported in primary studies, at least in aggregate form (ie, as frequencies or means). According to the Global Burden of Disease Study 2019 [47], complex interactions exist between sex or gender and age in terms of prevalence, deaths, and disability-adjusted life years of COPD. Thus, we aim to assess if systematic reviews consider any of the 3 variables either individually or as part of interactions on the outcomes of digital technologies in COPD. In addition, a number of other participant characteristics could be worth investigating in COPD, such as the age of onset [11], race [48], or education and socioeconomic status [49]. Furthermore, the focus on digital health technologies is also a reason to choose sex, gender, or age as the variables of interest in our overview. For example, the interest in and the actual use of digital health technologies in COPD may decline with age and depend on digital health literacy as is the case in the general population [19]. Studies assessing the acceptance and use of digital technologies often take into account sex, gender, or age as explanatory variables. For example, the gender gap in internet use (favoring males) was approximately 1.8% in 2020 [50]. However, when splitting the sample to assess older individuals (aged 75 years or older), a gender gap of 55% (favoring males) still persists [50]. When it comes to searching health-related information on the internet or using other technologies for health purposes, females outperform males [51], although internet use for health purposes declined with age (faster in females than in males). Indeed, while people with COPD had a positive attitude toward mobile health adoption for COPD management, especially the older participants who faced difficulties using such technologies owing to their age [52], we expect that sex, gender, or age could influence the outcomes of digital technologies for COPD. However, it is unclear if and how these variables are considered in systematic reviews of digital technologies for COPD.

### Strengths and Limitations

This protocol has been rigorously developed, and the electronic search syntax was iteratively tested and revised by an experienced database specialist. Nevertheless, we cannot exclude the possibility that some relevant systematic reviews in this new field may have been overlooked in our electronic search. Hence, a manual search for additional literature will be performed by screening the bibliographies of the included systematic reviews. The overview will also have further limitations. We have decided not to search the gray literature—this choice is guided by the general difficulty in assessing any financial interests associated with digital health technologies that may be present in gray literature. Our appraisal of systematic reviews will be based on AMSTAR 2 [26]. Another possible appraisal tool could be the risk of bias in systematic reviews (ROBIS), which was designed to evaluate the level of bias present within a systematic review [53]. So far, there are no clear recommendations as to which instrument is more suitable for overviews [54]. We have chosen AMSTAR 2 because the tool is easier to implement [55] and has a higher interrater reliability than ROBIS [30,56]. For the overall confidence rating required in the overview, AMSTAR 2 showed high agreement with ROBIS [30]. In addition, we will only include systematic reviews in English or German, which may further limit the relevant literature for this overview.

### Implications for Practice and Dissemination Plan

Evidence from the overview could be used to guide more individualized (sex-, gender-, or age-based) recommendations for the use of digital technologies by people with COPD. Considering the rapid technological advancement in the field of digital health technologies, the findings from the overview could be of interest for various stakeholder groups, including researchers, policy makers, health professionals, people with COPD, and companies that develop digital technologies for COPD. Therefore, the dissemination plan for this overview is to publish the findings in a peer-reviewed journal and present them at scientific conferences. We will also attempt to summarize the findings using a plain-language summary designed for the nonscientific community, which can be uploaded on our research profiles on the internet.

### Conclusions

There is a growing recognition that the influence of sex, gender, or age should be considered in reporting research designs and outcomes in the context of health care interventions. Our overview will help identify systematic reviews of various digital technologies for the treatment or monitoring of COPD. The most interesting aspect of the overview will be the ability to investigate if any systematic reviews considered the influence of sex, gender, or age on the outcomes of such digital technologies in COPD. Evidence from the overview could be used to guide more individualized (sex-, gender-, or age-based) recommendations for use of digital technologies by people with COPD.

## Abbreviations

AMSTAR2: A Measurement Tool to Assess Systematic Reviews, version 2
CCA: corrected covered area
COPD: chronic obstructive pulmonary disease
PICOS: population, intervention, comparison, outcomes, and study design
PRIOR: Preferred Reporting Items for Overviews of Reviews
PRISMA: Preferred Reporting Items for Systematic Reviews and Meta-Analyses
PROSPERO: International Prospective Registry of Systematic Reviews
RCT: randomized controlled trial
ROBIS: risk of bias in systematic reviews

## Data Availability

This overview of systematic reviews will be based on previously published data. All relevant data will be made available once the overview is complete.
